# Alcohol consumption among pregnant women in Brazilian capitals: How many, where, and who are they?

**DOI:** 10.31744/einstein_journal/2025AO0754

**Published:** 2025-02-26

**Authors:** Rinelly Pazinato Dutra, Giulia Piamolini Marques, Mariana Manfredi, Maria Eduarda Rodrigues Martins Chermont de Sá, Ane Priscila Konrad, Samuel de Carvalho Dumith

**Affiliations:** 1 Postgraduate Program in Health Sciences Faculdade de Medicina Universidade Federal do Rio Grande Rio Grande RS Brazil Postgraduate Program in Health Sciences, Faculdade de Medicina, Universidade Federal do Rio Grande, Rio Grande, RS, Brazil.; 2 Faculdade de Medicina Universidade Federal do Rio Grande Rio Grande RS Brazil Faculdade de Medicina, Universidade Federal do Rio Grande, Rio Grande, RS, Brazil.

**Keywords:** Pregnant women, Pregnancy, Alcohol drinking, Binge drinking, Risk factors, Health surveys, Surveys and questionnaires

## Abstract

**Objective:**

To analyze the prevalence and factors associated with alcohol consumption among pregnant women.

**Methods:**

This cross-sectional study examined pregnant women living in Brazilian capitals using information collected by the Noncommunicable Chronic Disease Risk Factor Surveillance System (Vigitel) between 2006 and 2021. This study calculated the prevalence of both excessive and any amount of alcohol consumption by pregnant women and their associations with sociodemographic variables. Multivariate analysis was performed with prevalence ratios based on Poisson crude and adjusted regressions; the threshold for statistical significance was set at p<0.05.

**Results:**

The sample comprised 4,734 pregnant women. The prevalence of any amount of alcohol consumption was 11.5% (95%CI = 9.8-13.6), and that of excessive consumption was 3.0% (95%CI = 2.1-4.2); both remained stable throughout the analyzed period. Being 35-54 years old and having low educational attainment were the main factors associated with both consumption patterns. Being Indigenous was highly associated only with excessive alcohol consumption, whereas being legally married and living in Northern Brazil were protective factors.

**Conclusion:**

These findings emphasize the need for focused preventive strategies targeting the most vulnerable groups to mitigate alcohol consumption and its associated risks during pregnancy.

## In Brief

Alcohol consumption during pregnancy poses serious risks to maternal and fetal health, making it a public health concern. This study sheds light on the sociodemographic disparities linked to alcohol use among pregnant women in Brazilian capital cities, highlighting vulnerable groups and regional variations. These findings underscore the urgent need to develop tailored preventive strategies to protect maternal and child health.

## Highlights

■ Any alcohol consumption during pregnancy: prevalence of 11.5%.

■ Excessive alcohol consumption during pregnancy: prevalence of 3.0%.

■ Risk factors: older age, low education, and Indigenous ethnicity.

■ Protective factors: being married and living in Northern Brazil.

## Graphical abstract

**Figure:**
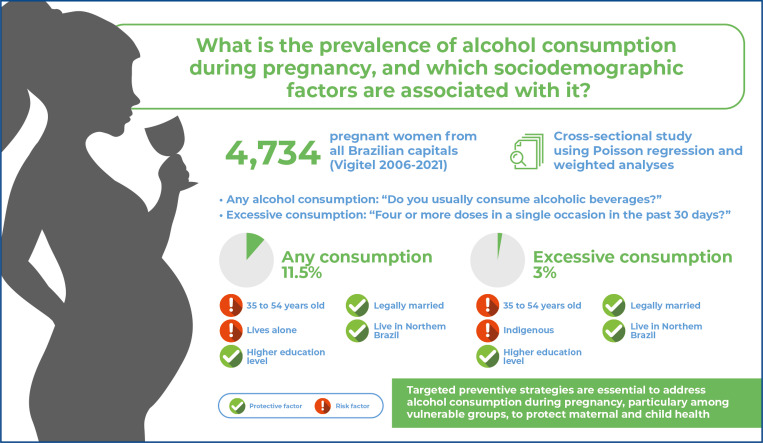

## INTRODUCTION

Alcohol is one of the most commonly consumed psychoactive substances worldwide.^1^Its consumption is a public health concern, because it causes significant metabolic and physiological changes.^2-4^ Alcohol consumption during pregnancy considerably increases the risk of adverse perinatal outcomes.^5^

The scientific literature has provided solid evidence of the harmful effects of alcohol consumption during pregnancy. Fetal alcohol spectrum disorder-which encompasses fetal alcohol syndrome (FAS), partial FAS, alcohol-related neurodevelopmental disorder, and alcohol-related congenital disabilities-is one of the main consequences assessed.^5-7^ Negative impacts on fetal development vary according to the amount of alcohol ingested by the mother, trimester, and maternal and fetal metabolic capacity, and other factors.^8^

While no guidelines have established a safe level of alcohol exposure during pregnancy, several health organizations, including the World Health Organization, American College of Obstetricians and Gynecologists, and Centers for Disease Control and Prevention, strongly recommend complete abstinence from alcohol consumption during pregnancy to prevent FAS and other developmental issues.^9-11^ Therefore, given the severe risks, it is essential to monitor and assess alcohol consumption in this population, as even moderate ingestion can have harmful effects on the fetus.^5^

Various systematic reviews and meta-analyses conducted overseas have investigated the prevalence of alcohol consumption during pregnancy.^8,12-14^ In Africa, it ranges from 2.2% to 15.5%,^14,15^ and in Latin America and the Caribbean, it ranges from 4.8% to 23.3%.^13^ In Brazil, most studies on alcohol consumption during pregnancy are conducted at local maternity hospitals, with prevalence ranging from 7.3% to 17.7%;^16-18^ however, a national hospital-based study conducted between 2011 and 2012 found a 14% prevalence of alcohol consumption during pregnancy, with 10% of women showing a presumptive diagnosis of inappropriate alcohol use during pregnancy.^19^

Despite the lack of consensus in the literature regarding the exact dosage that defines excessive alcohol consumption, its prevalence among pregnant women is 5.2% in the United States and 12.7% in Brazil.^18,20^ The main factors associated with alcohol consumption during pregnancy were being older than 30 years, being multiracial or Black, not living with a partner, being multiparous, having low educational attainment, and having low income.^18,19,21-23^

Given the risks posed to maternal health and fetal development, it is essential to investigate alcohol ingestion during pregnancy. Hence, this study aimed to fill an existing gap in the literature concerning alcohol consumption in pregnant women in Brazil, as most papers on this topic are local/regional investigations. Moreover, studies like this one can inform the development of strategies to plan, provide, and prioritize effective healthcare for this population.^12^

## OBJECTIVE

To analyze the prevalence and factors associated with excessive alcohol consumption among pregnant women living in Brazilian capital cities between 2006 and 2021.

## METHODS

This cross-sectional study used data from phone surveys conducted by a Noncommunicable Chronic Disease Risk Factor Surveillance System (Vigitel). These surveys are carried out annually, encompassing all Brazilian state capitals and the Federal District.

This study assessed data collected between 2006 and 2021 using a sample of pregnant women (n = 4,734) approached in the surveys. First, 5,000 phone numbers were selected randomly per city based on postal codes. After this, an additional screening process narrowed the sample to 200 phone numbers per city, excluding those that belong to companies, are out of service, or do not answer calls; therefore, six phone calls were made on different days and hours. Eligible phone numbers were then entered into another draw to select a household participant to answer the questionnaire. This person, who must be at least 18 years old, answered the self-reported questionnaire independently; other people were not allowed to participate at this stage.

The outcomes analyzed in the present study included excessive alcohol consumption and any amount of alcohol consumption among pregnant women. Alcohol consumption was assessed using the following question: “Do you usually consume alcoholic beverages?” Excessive alcohol consumption was defined based on a positive answer to the question, “In the last 30 days, have you consumed four or more servings of an alcoholic beverage on a single occasion?” In this case, a typical serving of an alcoholic beverage corresponded to a can of beer; a glass of wine; or a shot of cachaça (fermented sugarcane juice), whisky, or any other distilled alcoholic beverage.

The following sociodemographic variables were analyzed: age range (18-24, 25-34, 35-44, and 45-54 years), ethnicity (White, Black or Mixed-race, East Asian, and Indigenous), educational attainment (0-8, 9-11, 12 or more years at school), current marital status (single, legally married, domestic partnership for more than six months, widowed, separated/divorced, not reported), living alone (yes or no), and Brazilian region of residence (North, Northeast, Central-West, Southeast, or South). Black and multiracial ethnicities were grouped into a single category, Black or Mixed-race, due to the small sample sizes of these groups among the pregnant women. Further information on the methodological procedures is available in the Vigitel Annual Report.^24^

Descriptive statistical procedures were used to characterize the sample, using weighted relative and absolute frequencies. Cluster analysis with ranking, the method used by Vigitel, was used to assign a final post-stratification weight, considering consecutive comparisons between the estimated distribution of each sociodemographic variable and the total population of each municipality to align their distributions.^24^ Maps were created using ArcGIS software to depict the geographic distribution of both types of alcohol consumption (any quantity and excessive) according to different Brazilian regions. The multivariate analysis employed Poisson regression, both crude and adjusted, with all sociodemographic variables simultaneously included. All variables were retained in the final model because of their association with alcohol consumption during pregnancy (p<0.20), as they could act as potential confounders. Collinearity was assessed, with no significant collinearity detected. The results are presented as prevalence ratios (PR) along with 95% confidence intervals (95% CI); the statistical significance threshold was set at p<0.05. The analysis accounted for the sample weights and was adjusted for city clusters using the “vce” command. Temporal trends in alcohol consumption (both any and excessive consumption) between 2006 and 2021 were analyzed using the Prais-Winsten test with Cochrane-Orcutt transformation, accounting for the autocorrelation of residuals. All analyses were performed using Stata software, version 15.1 (College Station, TX, StataCorp LLC).

This study used public-domain data available from the Ministry of Health; as such, the need for ethical approval was waived for this study. Data were obtained from the interviewees with oral informed consent during phone calls. The study conducted by Vigitel was authorized by the National Human Research Ethics Committee of the Ministry of Health under the Certificate of Presentation for Ethical Appraisal (CAAE: 65610017.1.0000.0008; # 4324071).

## RESULTS

A sample of 4,734 pregnant women from all 26 Brazilian capitals and the Federal District was investigated between 2006 and 2021. The 25-34 age group predominated (54.9%). Regarding ethnicity, 39.6% were White, 39.7% were Black or Mixed-race, and the most common level of educational attainment was 9-11 years at school (47.0%). Most of these women lived with others in their homes (99.5%), and 48.1% were legally married. In addition, 43.8% of participants lived in Southeastern Brazil. As for the investigated outcome, 11.5% (95%CI = 9.8-13.6) reported consuming any amount of alcohol during pregnancy, while 3.0% (95%CI = 2.1-4.2) reported excessive alcohol consumption (Table 1).

**Table 1:** Characteristics of the sample of pregnant women included in the study

Variables	n (%)	
Age group (years)		
18-24	1,252 (26.5)	
25-34	2,597 (54.9)	
35-44	820 (17.3)	
45-54	65 (1.3)	
Ethnicity		
White	1,848 (39.6)	
Black or Mixed-race	1,851 (39.7)	
East Asian	941 (20.2)	
Indigenous	26 (0.5)	
Educational Attainment (years at school)		
0 to 8	1,069 (22.6)	
9 to 11	2,227 (47.0)	
12 or more	1,438 (30.4)	
Current marital status		
Single	1,521 (32.1)	
Legally married	2,276 (48.1)	
Domestic partnership	776 (16.4)	
Widowed	30 (0.6)	
Separated/Divorced	117 (2.5)	
Not reported	14 (0.3)	
Lives alone		
Yes	22 (0.5)	
No	4,712 (99.5)	
Region		
North	596 (12.6)	
Northeast	1,162 (24.6)	
Central-West	566 (11.9)	
Southeast	2,074 (43.8)	
South	336 (7.1)	
Alcohol consumption during pregnancy		
Yes	545 (11.5)	
No	4,189 (88.5)	
Excessive alcohol consumption during pregnancy		
Yes	143 (3.0)	
No	4,591 (97.0)	

**Figure 1:**
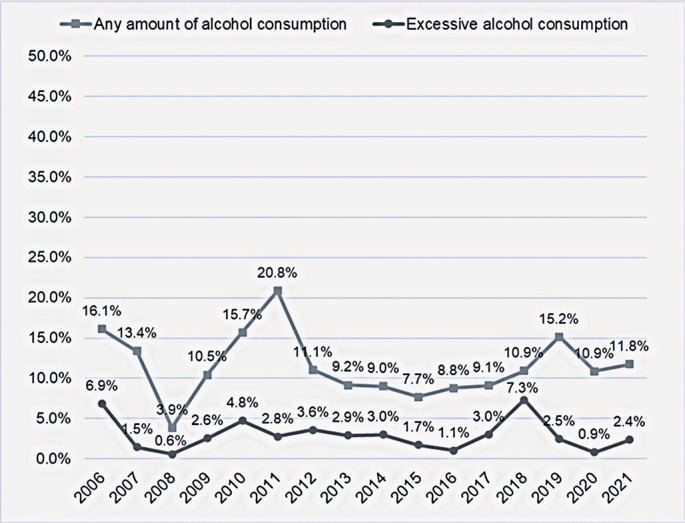
Temporal trend of the prevalence of excessive and any amount of alcohol consumption during pregnancy

Temporal analysis did not reveal a clear trend in terms of either excessive or any alcohol consumption during pregnancy. The prevalence of any amount of alcohol consumption ranged from 3.9% in 2008 to 20.8% in 2011 (β = 0.03; 95%CI= -0.67; 0.72; p=0.935), and that of excessive alcohol consumption ranged from 0.6% in 2008 to 7.3% in 2018 (β = 0.05; 95%CI= -0.18; 0.27; p=0.671), both with no linear pattern over time (Figure 1).

**Figure 2:**
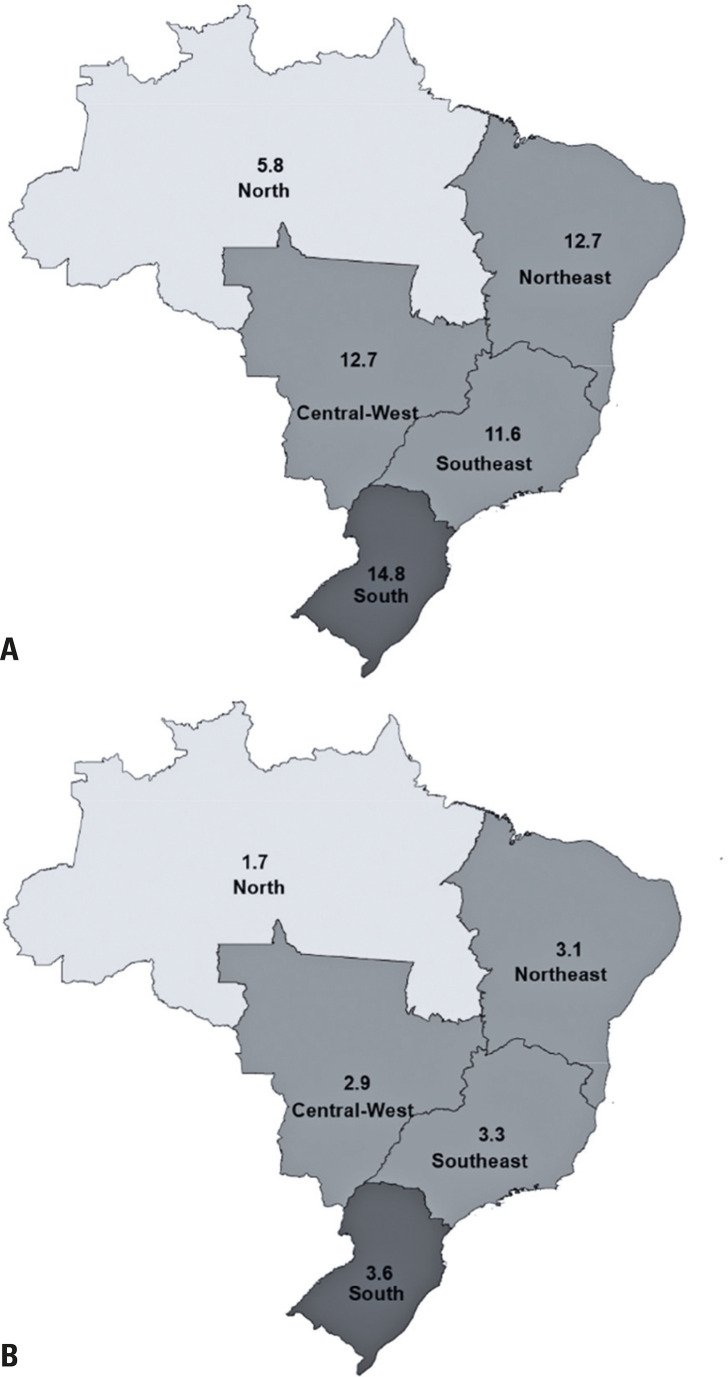
Distributions of (A) any amount of alcohol consumption and (B) excessive alcohol consumption by Brazilian region

Figure 2 presents the prevalence of drinking any quantity (Figure 2A) and excessively (Figure 2B) among pregnant women according to region. Alcohol consumption was higher among pregnant women residing in the Southern region, both at any quantity (14.8%) and in excess (3.6%). Conversely, the lowest prevalences were found in the Northern region, with 5.8% consuming any quantity and 1.7% excessively.

Table 2 shows the prevalence of the factors associated with excessive alcohol consumption and any amount of alcohol consumption during pregnancy. Among women who lived alone, the prevalence of alcohol consumption varied considerably, ranging from 5.8% in the North to 28.9%. Excessive consumption ranged from 0.0% among widows to 1.3% among married women and reached 15.0% among Indigenous women. Being older, having lower educational attainment, being single, and living in the North remained significantly associated with both types of consumption in the crude and adjusted multivariate analyses. Ethnicity is also strongly associated with excessive alcohol consumption.

**Table 2:** Prevalence and factors associated with both excessive and any amount of alcohol consumption during pregnancy

Variables	Consumption of any amount of alcohol			Excessive alcohol consumption			
	%	Crude	Adjusted*		%	Crude	Adjusted*
		PR (95% CI)	PR (95% CI)			PR (95% CI)	PR (95% CI)
Age group (years)							
18-24	12.3	1	1		3.7	1	1
25-34	9.1	0.74 (0.37;1.51)	0.92 (0.55;1.52)		1.6	0.41 (0.16;1.08)	0.58 (0.25;1.31)
35-44	17.1	1.39 (0.83;2.32)	1.74 (1.23;2.44)		5.9	1.59 (1.10;2.29)	2.20 (1.58;3.06)
45-54	22.4	1.82 (0.92;3.63)	1.97 (1.36;2.87)		10.5	2.79 (0.49;16.0)	3.00 (1.11;8.13)
Ethnicity							
White	11.4	1	1		2.2	1	1
Black or Mixed-race	11.8	1.04 (0.82;1.31)	0.96 (0.77;1.19)		3.4	1.56 (1.00;2.45)	1.25 (0.80;1.98)
East Asian	11.1	0.98 (0.61;1.56)	0.91 (0.51;1.63)		3.2	1.45 (0.84;2.50)	1.16 (0.73;1.84)
Indigenous	20.9	1.84 (0.77;4.41)	1.58 (0.71;3.50)		15.0	6.83 (1.88;24.8)	5.54 (1.96;15.6)
Educational attainment (years)							
0 to 8	17.4	1	1		6.1	1	1
9 to 11	9.4	0.54 (0.37;0.85)	0.57 (0.36;0.90)		2.1	0.35 (0.19;0.64)	0.39 (0.22;0.70)
12 or more	10.5	0.60 (0.36;1.00)	0.60 (0.37;0.98)		2.2	0.36 (0.19;0.68)	0.35 (0.15;0.84)
Current marital status							
Single	14.9	1	1		4.4	1	1
Legally married	8.2	0.55 (0.32;0.94)	0.54 (0.35;0.84)		1.3	0.29 (0.15;0.56)	0.31 (0.15;0.68)
Domestic partnership	15.2	1.02 (0.67;1.53)	1.07 (0.72;1.60)		5.2	1.18 (0.68;2.03)	1.23 (0.34;6.93)
Widowed	9.9	0.67 (0.17;2.56)	0.49 (0.15;1.60)		0.0	-	-
Separated/Divorced	10.8	0.73 (0.21;2.47)	0.68 (0.24;1.97)		5.9	1.34 (0.26;6.86)	1.53 (0.34;6.93)
Lives alone							
No	28.9	1	1		9.8	1	1
Yes	11.4	2.51 (1.44;4.39)	2.03 (1.21;3.43)		3.0	3.29 (1.10;9.83)	2.25 (0.89;5.70)
Region							
Southeast	11.6	1	1		3.3	1	1
North	5.8	0.50 (0.35;0.72)	0.50 (0.34;0.75)		1.7	0.52 (0.36;0.75)	0.52 (0.34;0.80)
Northeast	12.7	1.09 (0.86;1.38)	1.09 (0.83;1.43)		3.1	0.92 (0.71;1.20)	0.93 (0.72;1.20)
Central-West	12.7	1.09 (0.86;1.37)	1.19 (0.92;1.55)		2.9	0.87 (0.39;1.96)	1.19 (0.51;2.77)
South	14.8	1.27 (0.89;1.82)	1.27 (0.98;1.65)		3.6	1.07 (0.58;1.99)	1.29 (0.59;2.83)

* Mutually adjusted for all covariates in the table.

The adjusted analysis verified that pregnant women aged 35- 44 years and 45-54 years (respectively, PR = 1.74, 95%CI= 1.23-2.44; and PR = 1.97, 95%CI= 1.36-2.87) and who lived alone (PR = 2.03; 95%CI= 1.21-3.43) were more likely to consume any amount of alcohol during pregnancy. In contrast, pregnant women who had attended school for 9-11 years (PR = 0.57; 95%CI = 0.36-0.90) and 12 or more years (PR = 0.60; 95%CI = 0.37-0.98), who were legally married (PR = 0.54; 95%CI = 0.35-0.84), and lived in the North (PR = 0.50; 95%CI= 0.34-0.75) were less likely to consume any amount of alcohol during pregnancy (Table 2).

The likelihood of excessive alcohol consumption during pregnancy doubles for women aged 35-44 years (PR = 2.20; 95%CI= 1.58-3.06) and triples for those aged 45-54 years (PR = 3.00; 95%CI= 1.11-8.13). Moreover, being Indigenous increased the likelihood of excessive alcohol consumption 5.54-fold (95%CI= 1.96-15.6). As in any amount of alcohol consumption, educational attainment was a predictive factor, decreasing the likelihood by 61% (PR = 0.39; 95%CI= 0.22-0.70) for pregnant women who attended school for 9-11 years and 65% (PR = 0.35; 95%CI= 0.15-0.84) for those who attended it for 12 or more years. Legally married women (PR = 0.31; 95%CI= 0.15-0.68) and North region residents (PR = 0.52; 95%CI= 0.34-0.80) were likewise less likely to consume alcohol excessively during pregnancy (Table 2).

## DISCUSSION

This study aimed to analyze the prevalence of alcohol consumption among pregnant women and factors associated with it in Brazil between 2006 and 2021. We verified that one out of every ten investigated pregnant women reported having consumed any amount of alcohol, while one in every 30 reported having done so excessively during pregnancy.

When associated factors were considered, we found that alcohol consumption was more frequent among older pregnant women and those who lived alone, whereas excessive alcohol consumption was more prevalent among older and Indigenous pregnant women. In addition, the likelihood of either consumption pattern was lower among pregnant women with a higher educational attainment, those who were legally married, and those who lived in Northern Brazil.

Regarding alcohol consumption in the general female population, data from population surveys, such as the National Health Survey and the National Survey on Drug Use by the Brazilian Population, have investigated alcohol consumption in the general population over the last 12 months, finding prevalence rates of 17% and 35%, respectively, for alcohol consumption among women and 9.2% and 9.5%, respectively, for excessive consumption.^25,26^ Although these data do not specifically refer to pregnant women, they are relevant for contextualizing the prevalence of alcohol consumption among women in general, as alcohol consumption before pregnancy appears to be a strong predictor of consumption during the gestational period.^27^

Based on the population information made available by the Brazilian Institute of Geography and Statistics^28^ and considering the prevalence of outcomes in this study, it is estimated that approximately 44,500 pregnant women who live in the Brazilian capital cities consume any quantity of alcohol, and 11,600 of them do so excessively during pregnancy. These results reveal a worrying situation, since alcohol consumption during pregnancy is related to adverse fetal health outcomes.^5,7,29^

Alcohol is a teratogenic agent that causes hemodynamic changes and compromises blood flow to the placenta.^21^ The most relevant adverse perinatal effects include the greater likelihood of low birth weight, which occurs due to peroxidative processes that impair organogenesis and hinder the provision of nutrients. This results in respiratory distress and difficulties with breastfeeding.^21,30,31^

Although primary healthcare systems generally provide comprehensive prenatal coverage,^32,33^ this study identified a similar profile among women who consumed alcohol during pregnancy and those who did not attend or receive adequate prenatal care, as verified by Cabral et al and Rosa et al.^20,34^This relationship suggests that instructions given by health professionals associated with healthier habits adopted by pregnant women are crucial aspects of prenatal care to reduce adverse perinatal outcomes and promote health education, family planning, and later follow-up of children in postnatal care.^4,34^

Older age appears to be a predictive factor for greater alcohol consumption during pregnancy, both in any amount and excessively. Evidence suggests that older women are more likely to maintain habits established throughout their lives, such as alcohol consumption during pregnancy, which, combined with greater parity, may result in a false feeling of safety and lead to other risky behaviors.^18,35^ Furthermore, alcohol consumption in younger women is characterized by intense episodes on a few weekly occasions instead of frequent consumption. This occasional consumption pattern makes it more challenging to identify the problem early and ascertain the associated harmful habits.^36,37^

The present study corroborates existing evidence that pregnant women with lower educational attainment are more susceptible to potential risks to maternal and fetal health, including alcohol consumed in any amount or excessively.^18,19,22,23^Women with higher educational attainment tended to be more familiar with pregnancy-related instructions from health agencies. Therefore, they are more likely to make decisions based on reproductive planning and to have healthier habits, including abstinence from alcoholic beverages during pregnancy.^3,17^

The study found that married pregnant women were less likely to consume alcohol than single ones, either in any amount or in excess. Similarly, Cabral et al. found that alcohol consumption was higher among women without partners.^20^Partners who are present during pregnancy can play a significant role,^38^ as active and conscious paternity helps in both reproductive planning and care of the baby.^39^ Likewise, the lack of an adequate support network may leave pregnant women responsible for bearing and raising their children alone, which in turn leads to stress. Thus, the absence of a fixed partner may have a negative impact on prenatal, perinatal, and postnatal care, possibly exposing pregnant women to risks, such as alcohol consumption.^21,36^

The results also showed lower alcohol consumption among pregnant women in the North region, which has been identified in the literature regarding alcohol consumption both in the general population and among pregnant women.^19,25,26,37,40^The population and regional characteristics also contribute to such results, as this region is less urbanized, making it more difficult to transport products and inputs to those states.^40^

Lastly, pregnant Indigenous women had the highest excessive alcohol consumption among all ethnicities analyzed. Although various studies have addressed such use by this community,^41-44^ the prevalence of consumption by pregnant indigenous women has been poorly discussed. When explaining this result, one must understand Indigenous people in their collective and social context, analyzing the historical processes that involve European colonization, marked by power and domination struggles that changed consumption patterns^43^ and thus helped increase alcohol intake. Unfortunately, these events still affect the daily lives of Indigenous women, who remain marginalized and whose health during pregnancy is neglected.^45^

This study has several strengths and limitations. First, the Vigitel data enables health monitoring and surveillance, as it is carried out yearly and includes a representative sample of the inhabitants of Brazilian capitals. This study also used data collected over 16 years, which considerably increased its analytical power. Moreover, this study is relevant to the very topic it addresses, since any amount of alcohol consumption during pregnancy can impact maternal-child health. However, the limitations of the study must be considered, including the fact that Vigitel data is limited to Brazilian capitals and homes with landline phones. In addition, self-reported questions can lead to biased results that poorly represent the reality of pregnant women, underestimating investigated outcomes. The prevalence found, though substantial, may be even greater in reality, as many pregnant women omit information when asked about their alcohol use for fear of either being rebuked or having their identity disclosed. Furthermore, the study did not assess other critical sociodemographic variables, such as social support, personal psychiatric history, and intimate partner violence, which are not collected by Vigitel and could potentially act as residual confounders.

## CONCLUSION

In conclusion, despite the prevalence remaining stable throughout the analyzed years, a considerable proportion of pregnant women reported alcohol consumption in any quantity. Additionally, a smaller but concerning share of women reported excessive consumption during pregnancy. These findings underscore the urgency of targeted preventive approaches, particularly towards pregnant women with lower educational attainment and those of Indigenous origin, who emerged as the most vulnerable groups, aiming to mitigate alcohol consumption and the underlying risks during the gestational period.
